# Indirect effect of 7-valent and 13-valent pneumococcal conjugated vaccines on pneumococcal pneumonia hospitalizations in elderly

**DOI:** 10.1371/journal.pone.0209428

**Published:** 2019-01-16

**Authors:** Irina Kislaya, Ana Paula Rodrigues, Mafalda Sousa-Uva, Verónica Gómez, Paulo Gonçalves, Filipe Froes, Baltazar Nunes

**Affiliations:** 1 Departamento de Epidemiologia, Instituto Nacional de Saúde Doutor Ricardo Jorge, Lisboa, Portugal; 2 Centro de Investigação em Saúde Pública, Escola Nacional de Saúde Pública, Universidade Nova de Lisboa, Lisboa, Portugal; 3 Departamento de Doenças Infecciosas, Instituto Nacional de Saúde Doutor Ricardo Jorge, Lisboa, Portugal; 4 Thorax Department, Centro Hospitalar Lisboa Norte, Lisboa, Portugal; Harvard School of Public Health, UNITED STATES

## Abstract

**Background:**

Pneumonia is one of the leading causes of mortality and has a high burden in morbidity. In Portugal, 7-valent pneumococcal conjugated vaccine (PCV) was used since 2001 and PCV10/13 since 2009, being the last introduced into the National Immunization Program in 2015.

**Methods:**

We conducted an ecological study to evaluate the impact of PCV7 and PCV13 on pneumococcal pneumonia (PP) hospitalizations in adults aged 65 years or more in Portugal. National hospital discharge registry data from 1998/99 to 2015/16 were used, and PP hospitalization was defined as any hospitalization coded in primary diagnosis as 481 (ICD-9-CM) or J18 (ICD-10-CM). Poisson regression models adjusted for seasonality, influenza-like illness and allowing for overdispersion was used to estimate annual average change of PP hospitalization rate. To assess PP hospitalization trends before and after PCV7 and PCV13 introduction interrupted time series analysis was performed.

**Results:**

In 1998/99 PP hospitalization rate was 7.0 per 10,000 inhabitants, varying between 3.2 (females, 65–74 years) to 20.7 (males, +85 years), and annually increasing by 16% during the pre-PCV7 period. Statistically significant reduction of 14% per year in PP hospitalization rate was observed after PCV7 introduction. Between 2004/05 and 2009/10 PP hospitalization rate decreased annually by 4% and after PCV13 introduction by 11% per year. In 2015/16 we found an overall reduction of 2.9 (CI 95%: 2.7; 3.1) PP hospitalizations per 10,000 inhabitants (598 hospitalizations) attributable to PCV13, varying from 2.2 (CI 95%: 1.3; 3.1) (female, 65–74 years) to 5.6 (CI 95%: 3.8; 7.5) (female, +85 years).

**Conclusions:**

Our results suggest that introduction of both PCV7 and PCV13 vaccines resulted in the reduction of PP hospitalizations rates among older adults.

## Introduction

Pneumonia is one of the leading causes of mortality in extreme young and older ages worldwide [1] and presents a high burden in morbidity, namely among elderly [2]. *Streptococcus pneumoniae* is being referred as the main aetiological agent of pneumonia, varying its frequency accordingly different settings [2]. Though *Streptococcus pneumoniae* is responsible for different infection presentations, pneumonia (bacteremic or non-bacteremic) is the most frequent form of pneumococcal infection among adults [3]. While 13-valent pneumococcal conjugated vaccine (PCV13) has been associated to a reduction of vaccine serotypes of Community Acquired Pneumonia [4], efficacy of polysaccharide vaccine (PPSV23) against pneumonia in elderly remains controversial [5]. Since the World Health Organization had recommended to introduce PCV into childhood immunization programs in 2000, there is a consistent and grown evidence of pneumococcal disease reduction in vaccinated children, but also in other population subgroups not directly targeted by vaccination programs [6]. Such decrease can be attributed to indirect protection (herd effect) through decreasing of vaccine serotype carriage and subsequent reduction in disease transmission among non-vaccinated groups [7]. In addition to the direct protection achieved through child immunization, this advantageous herd effect has been highlighted in public health field as a way to protect more vulnerable groups. Older adults can be those most beneficed given the high incidence of pneumococcal infection and their lower response to pneumococcal vaccines due immunosenescence [8,9].

During the last two decades interrupted time series analysis were extensively used to evaluate population-level impact of PCV [6,9] and the reduction of Invasive Pneumococcal Disease (IPD) in children and adults is well documented in the United States of America (USA) [8,10,11], as well as in several European countries [9,12]. It is plausible that the indirect effect of PCV on IPD and on non-invasive Pneumococcal Pneumonia (PP) differs, as these different forms of pneumococcal disease are caused by different serotypes [13,14]. Notwithstanding to our knowledge, less is known regarding PCV impact on adult PP [9] and literature provides contradictory results on the issue. Simonsen et al had shown a significant reduction of PP hospitalizations in elder adults after PCV7 and PCV13 immunization programs implementation in USA [8,10]. Similar conclusions were reached by Menzies et al [15] for Australian elder adults after PCV7 introduction. More recently, a significant decrease in all-cause pneumonia, but not in PP hospitalizations in adults aged 65 years and more after PCV10 introduction was shown in Finland [16]. This indirect effect has not been seen in Scotland [17] and in UK, where the most recent study had found an increasing trend in PP hospitalizations in adults aged 65 years and more after PCV13 introduction [18], despite the reduction of PP incidence reported in the 5 following years after PCV13 introduction [19]. The variability on population structure and epidemiological figures across European countries reinforces the need of additional research to provide a more comprehensive picture of direct and indirect effect of PCV13 to support public health planning at European and country level.

Thus, we considered that analyzing PP hospitalizations trends in Portugal may provide new insights on the issue and contribute to improve the growing body of evidence, since Portugal is one of those countries that recently introduced PCV13 into the National Immunization Program (NIP) after 14 years of widely recommended use of PCV7 and PCV13 in healthy children.

Among Portuguese adults, PCV13 serotypes increased until 2008 in both IPD and non-invasive PP, mainly due to the six additional serotypes not included in PCV7, indicating a probable serotype replacement after PCV7 use [13,14] and, therefore, contributing to a higher potential for prevention by using PCV13. However, vaccine impact at population-level was not previously assessed. To test this hypothesis, we conducted an ecological study, which assessed the indirect effect of pneumococcal vaccines (PCV7 and PCV13) on PP hospitalization rate among adults aged 65 years and more in Portugal.

## Methods

### Data source, study population and case definition

A retrospective time-series study was conducted using information recorded in the national hospital discharge database from 1 July 1998 to 30 June 2016 (18 epidemiological years).

National Hospital Discharge Database covers all public hospitals in mainland and includes data on diagnosis, external causes, procedures, related-costs, length of stay and outcome discharge for all episodes of care occurred at hospital level and patients demographics data as well. Diagnoses, procedures and external causes were coded using the International Classification of Diseases (ICD) - 9^th^ Revision Clinical Modification (ICD-9-CM) [20] until 1^st^ January 2016 and ICD-10-CM [21] after that date.

Study population encompassed individuals aged 65 years or more, residents in mainland Portugal. PP hospitalization was defined as an episode with a principal diagnosis coded as 481 (ICD-9-CM) or J13 (ICD-10-CM). We considered only episodes with hospital stay length greater than 24 hours. Episodes with sex or age variables missing were excluded, as well as those with recorded age higher than 105 years (n = 753).

Official estimates of the resident population in mainland Portugal for each study year [22], stratified by sex and age group (65–74, 75–84, 85 years or more) were used as denominators. Monthly hospitalization rates per 10,000 inhabitants were stratified by sex and age group.

### Vaccines and vaccine coverage

**Fig 1:**
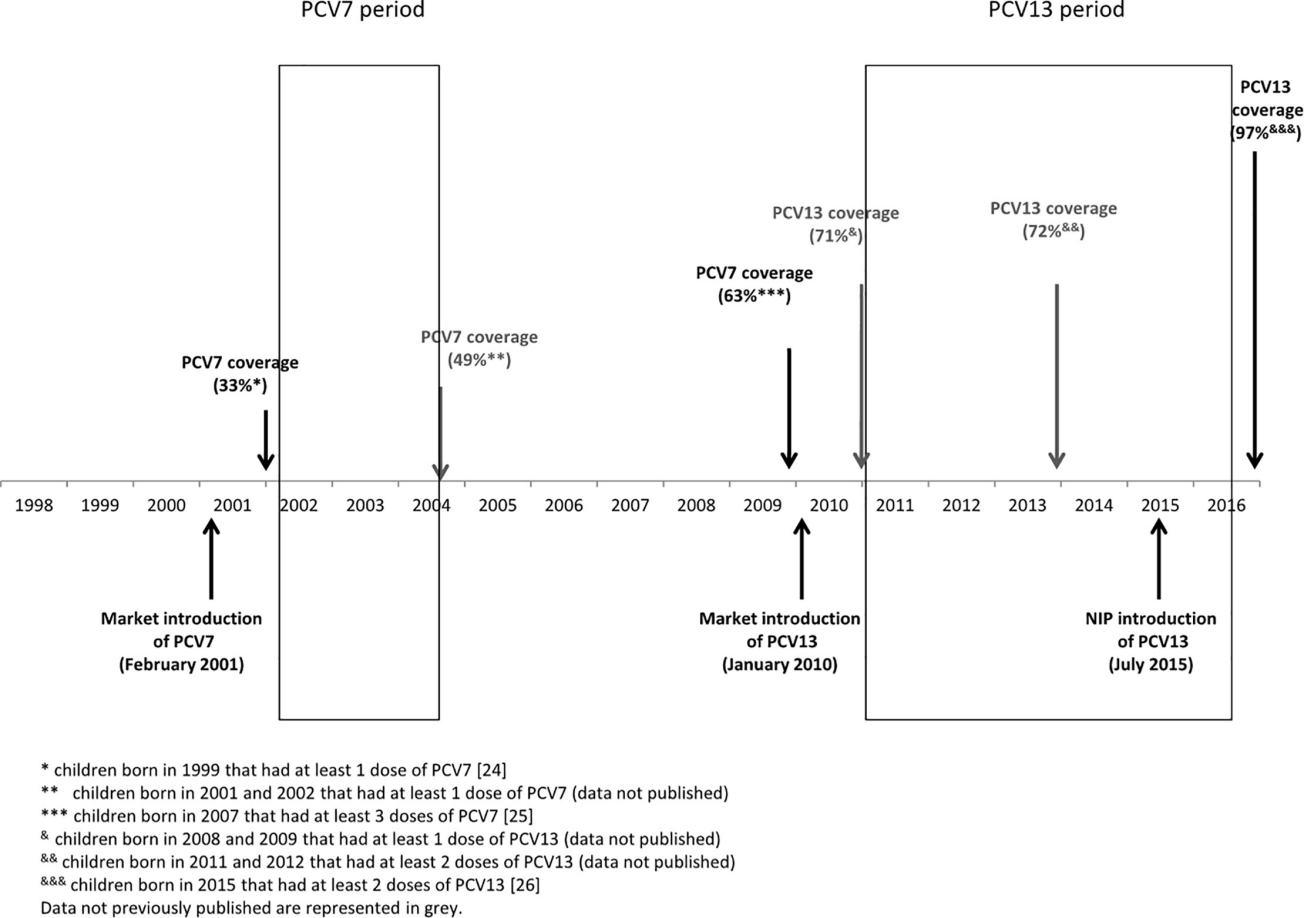
Schematic representation of pneumococcal conjugated vaccines use and coverage in Portugal.

PCV13 was introduced into the NIP for all children under 2 years old using a 2+1 vaccination scheme (at 2 and 4 months and booster at 12 months) in July 2015, without catch up campaign [23]. PCV were available at the national market soon after their European license—PCV7 in February 2001, PCV10 in April 2009 and PCV13 in January 2010—and their use was largely recommended by paediatricians for all healthy children, allowing to reach moderate PCV coverage even before its introduction into the NIP. PCV7 coverage ranged from 33% in 2001 [24] to 63% in 2009 [25], being around 50% in almost all years during which PCV7 was used (unpublished data). PCV13 coverage was around 70% between 2011 and 2015, and after its introduction into the NIP reached 97% [26] (Fig 1). PCV13 and PPSV23 in elderly didn´t reach 10% of coverage (unpublished data).

### Statistical analysis

Annual PP hospitalization rates were estimated by epidemiological year (1 July to 30 June). Interrupted time-series analysis was performed to compare trends in hospitalization rates before and after PCV7 and PCV13 use in children.

Pre-PCV7 period included 31 time points from July 1998 to January 2001. Following twelve points (February 2001 to January 2002) were considered as transitional period and excluded from the analysis. PCV7-period included 41 time points from February 2002 to June 2005.

Pre-PCV13 period comprised 66 time points from July 2004 to December 2009; following twelve points (January 2010 to December 2010) were excluded from the analysis and considered transitional period; and PCV13 period included 66 time points from January 2011 to June 2016.

Poisson regression models with correction for overdispersion were used to assess trends in hospitalization rates separately for each age group, for both male and female. Models assumed the following form:

$$logE(Y_{t})=\beta_{0}+\beta_{1}T_{t}+\beta_{2}X_{t}T_{t}+\beta_{3}\sin\left(2\pi T_{t}/12\right)+\beta_{4}(\cos2\pi T_{t}/12)+\beta_{5}I_{t}+log({Pop}_{t}),$$

where *Y*_*t*_ is a monthly count of pneumonia hospitalizations in each sex/age group stratum; *β*_0_ is a baseline rate *T*_*t*_ is time in months since the start of the study; *X*_*t*_ is a dummy variable representing the vaccine use (0 in pre-vaccine period, 1 otherwise); *β*_1_ is a slope of the outcome variable until vaccine use; *β*_2_ is the change in the slope after vaccine use. To adjust for seasonality, models included linear combination of sine and cosine functions. Influenza-like illness incidence rates (*I*_*t*_) obtained from the Portuguese National Influenza Surveillance System were also included in the model to account for effect of influenza epidemics and other respiratory viruses on PP hospitalizations [27]. Logarithm of population in time *t* (*Pop*_*t*_) was included as offset. Models fitted for the whole population were additionally adjusted for sex and age group. Models assumptions were verified using graphical analyses of residuals and checked for autocorrelation and partial autocorrelation.

Annual percentage change of pneumonia hospitalization rates in pre and pos-vaccine periods was calculated as 100%*(RR-1), where RR (Rate Ratio) was estimated for 12 months as *exp*(12 * *β*_2_).

The indirect effect of PCV7/PCV13 was obtained by subtracting the predicted PP hospitalization rate for each studied vaccine period from the expected PP hospitalization rate in the absence of vaccine use. Separate models were fitted to each vaccine, age group and sex. The number of PP hospitalizations prevented by vaccine per year was estimated multiplying the absolute rate difference by the population size.

### Control conditions and sensitivity analysis

To assess whether observed trends were due to changes in secular trends in hospital admissions, changes in other pneumonia risk factors or changes in coding practice, the following additional outcomes were used:

1. All-cause hospitalizations excluding those due to all-cause pneumonia;
2. Other bacterial pneumonia (ICD-9-CM: 482.0, 482.1, 482.2; ICD-10-CM: J14, J15.0, J15.1). This control group was selected since it includes other pneumonia frequently observed among elder adults, which share risk factors with PP and have similar hospital admission likelihood, but are not prevented by pneumococcal vaccines;
3. PP in any first seven diagnoses;
4. PP in primary diagnosis combined with sepsis/bacteremia in primary diagnosis (CID-9-CM: 038; 995.91; 995.92; 785.92; 790.7; CID-10-CM: A40; A41; R65.20; R65.21; R78.81) [28] and PP as secondary diagnosis (up to six diagnoses).

All data were anonymized, thus informed consent was not required. Ethical Committee of the *Instituto Nacional de Saúde Doutor Ricardo Jorge* approved the study protocol.

## Results

### Characteristics of PP hospitalizations

Between July 1998 and June 2016, 5,847,063 hospitalizations of individuals aged 65 years and more were registered, from which 22,832 (0.39%) were coded as PP on primary diagnosis. The average (and median) length of stay for PP hospitalization remained relatively stable ranging between 11.6 days (10.0 days) in 1998/99 and 11.2 days (9.0 days) in 2015/16, while the median age of PP patients increased from 77 to 81 years.

### Trends in PP hospitalization rate

In 1998/99, the overall PP hospitalization rate was 7.0 per 10,000 inhabitants, markedly varying by age. Highest rates were observed among those aged 85 years or more, in both male (20.7 per 10,000) and female (14.5 per 10,000). Males presented always a higher rate than females (Table 1).

**Table 1:** Annual PP rate per 10,000 inhabitants stratified by sex and age group between 1998/99 and 2015/16, Portugal mainland.

Year	Total	Male			Female		
		65–74	75–84	≥ 85	65–74	75–84	≥ 85
1998/99	7.0	6.3	12.6	20.7	3.2	6.4	14.5
1999/00	7.3	5.1	13.5	28.1	3.4	7.8	13.3
2000/01	8.0	6.7	13.1	30.3	3.3	8.4	14.8
2001/02	9.5	6.9	17.4	34.0	4.3	8.7	20.9
2002/03	8.7	6.7	15.0	34.6	3.8	8.3	18.9
2003/04	10.2	7.2	18.7	38.6	4.0	9.7	23.4
2004/05	8.9	6.4	16.0	39.3	3.1	8.7	18.8
2005/06	7.4	5.4	14.5	28.6	2.5	6.7	15.5
2006/07	8.0	5.8	14.3	29.0	3.0	7.1	18.2
2007/08	7.5	5.3	12.8	29.1	2.2	6.6	20.5
2008/09	9.0	6.4	15.3	30.2	3.3	8.1	21.1
2009/10	7.1	5.3	11.7	27.7	2.3	6.4	15.8
2010/11	6.8	5.2	10.9	25.3	2.5	5.5	14.7
2011/12	6.8	4.0	10.6	27.5	2.3	5.9	16.7
2012/13	4.0	3.3	6.2	15.5	1.0	3.4	8.9
2013/14	4.2	2.9	5.8	15.6	1.2	3.8	11.1
2014/15	4.2	2.7	6.4	16.2	1.4	3.5	9.2
2015/16	4.0	2.6	5.7	15.5	1.3	3.8	8.5

In pre-PCV7 period (July 1998—January 2001) the overall PP hospitalization rate increased in average by 16% per year (RR = 1.16). For both males and females, statistically significant increasing trends were observed in all age groups, with the highest increase among males aged 75–84 years old (Table 2).

**Table 2:** Annual trends in PP hospitalization rate by sex and age group, before and after PCV7 and PCV13 use, Portugal mainland.

	Pre-PCV study period		PCV study period		Test for change in trend
	RR	CI 95%	RR	CI 95%	p-value
**PCV7**					
Total	**1.16**	[1.12; 1.21]	**0.86**	[0.80; 0.93]	0.001
*Male*					
65–74	**1.12**	[1.02; 1.24]	0.87	[0.72; 1.04]	0.126
75–84	**1.20**	[1.09; 1.33]	**0.84**	[0.70; 0.99]	0.044
85+	**1.17**	[1.05; 1.31]	0.93	[0.76; 1.12]	0.441
*Female*					
65–74	**1.19**	[1.09; 1.30]	**0.76**	[0.64; 0.90]	0.001
75–84	**1.14**	[1.05, 1.25]	0.89	[0.77, 1.03]	0.130
85+	**1.19**	[1.03; 1.36]	0.90	[0.71; 1.14]	0.388
**PCV13**					
Total	**0.96**	[0.94; 0.97]	**0.89**	[0.86; 0.91]	<0.001
*Male*					
65–74	0.97	[0.93; 1.00]	**0.86**	[0.79; 0.93]	<0.001
75–84	**0.95**	[0.91; 0.98]	**0.89**	[0.82; 0.96]	0.004
85+	**0.95**	[0.91; 0.99]	0.94	[0.86; 1.03]	0.195
*Female*					
65–74	0.98	[0.93; 1.04]	**0.81**	[0.72; 0.91]	<0.001
75–84	**0.95**	[0.91; 0.99]	**0.90**	[0.82; 0.98]	0.014
85+	0.97	[0.93; 1.01]	**0.90**	[0.83; 0.97]	0.007

***Notes*:** RR: Rate Ratio. Statistically significant results are in bold. Models shown no significant deviation from assumptions.

**Fig 2:**
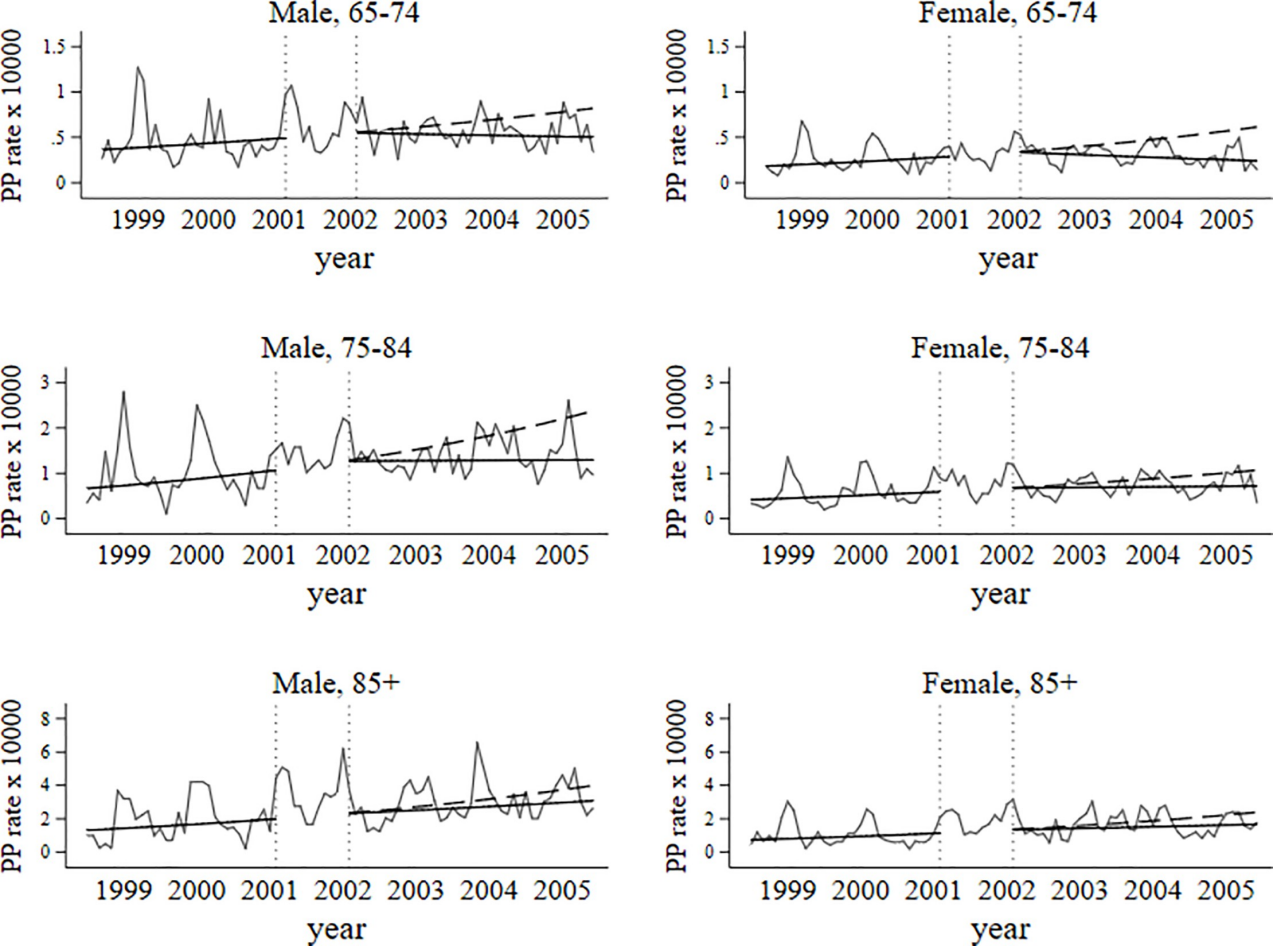
Trends in PP hospitalization rates before and after PCV7 use.

After PCV7 use (February 2002 to June 2005) PP hospitalization rate declined in two population subgroups, in women aged 65–74 and in men aged 75–84 years old. The observed decreasing trend was statistically significant (Table 2, Fig 2) also for overall sample, representing 14% reduction per year.

During pre-PCV13 period (July 2004 to December 2009) overall PP hospitalization rate declined on average by 4% per year (Table 2). A more pronounced and statistically significant decrease trend (11% per year) was observed during PCV13-period.

Considering sex and age-specific results, in pre-PCV13 period no significant trends in annual PP hospitalization rate were observed for 65–74 age group, both males and females. But in PCV13 period this age group presented the highest reduction in both sexes: 14.2% per year for males and 19.2% per year for females.

In pre-PCV13 years, for 75–84 age group we found a similar rate reduction for males and females (5.3% and 5.0% per year, respectively). After PCV13 use, PP hospitalization rate declined at a faster rate in both sexes (11.2% in males and 10.3% in females).

**Fig 3:**
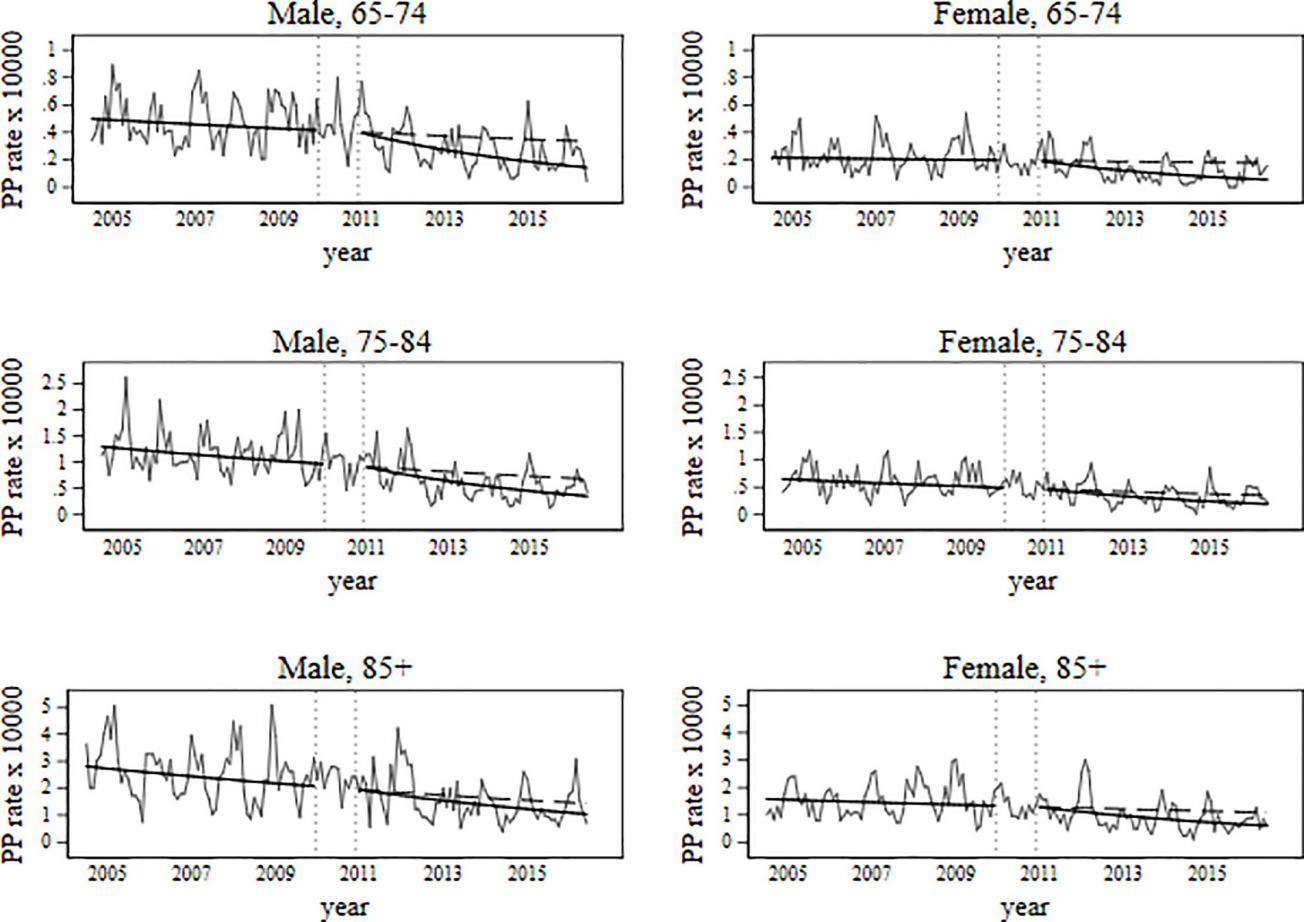
Trends in PP hospitalization rates before and after PCV13 use.

In the oldest age group different trends were observed between sexes. While in males PP hospitalization rate decreased by 5.5% per year in pre-PCV13, remaining unchanged in PCV13 years, in females no significant trend was observed in pre-PCV13 period, whereas in PCV13 period a rate reduction of 10.3% per year was observed (Fig 3).

At the end of the study period, PP hospitalization rate among those aged 65 years and more was significantly below than expected in all age groups and in both sexes. From our modeling analysis of changes in PP hospitalizations attributable to PCV7 and PCV13, Table 3 shows the prevented number of PP hospitalizations due to PCV7 and PCV13 and the absolute rate difference attributable to PCV7 and PCV13. Thus, in 2004/05 and in 2015/16 we found an overall reduction of 4.6 and 2.9 hospitalizations per 10,000 inhabitants, respectively, attributable to PCV7 and PCV13, which translated in 809 and 598 fewer hospitalizations (Table 3, Figs 2 and 3), respectively. Whilst the largest relative annual decrease in PP hospitalization rate in PCV13 period was found in the youngest age group (Table 2), the absolute rate difference increased with age (Table 3), reflecting the increasing burden of PP with age (Table 1).

**Table 3:** Prevented hospitalizations and absolute rate differences between expected and predicted hospitalizations rates at the end of PCV7 and PCV13 periods.

	Expected annual rate in absence of vaccine use (/10^4^)	Predicted annual rate (/10^4^)	Absolute rate difference and CI 95% (/10^4^)	Prevented number of hospitalizations (CI 95%)
**PCV7 (2004/05 year)**				
*Total*	13.8	9.1	4.6(4.2; 5.1)	809(736; 832)
Male				
65–74	10.0	6.6	3.4(1.6; 5.3)	152 (72; 230)
75–84	28.0	16.8	11.3 (6.3; 16.3)	267(149; 385)
85+	46.5	37.6	8.9 (-0.7; 18.4)	44(-4; 91)
Female				
65–74	7.3	3.2	4.1 (2.8; 5.3)	220 (154; 286)
75–84	12.2	8.8	3.4 (1.6; 5.2)	122 (58; 187)
85+	24.8	20.1	4.7 (-0.8, 10.3)	51 (-9; 112)
**PCV13 (2015/16 year)**				
Total	6.4	3.5	2.9 (2.7; 3.1)	598 (560; 635)
*Male*				
65–74	5.1	2.3	2.8 (1.9; 3.6)	130 (90; 170)
75–84	8.8	4.8	4.0 (2.7; 5.3)	121 (81; 161)
85+	18.7	13.9	4.8 (1.7; 7.9)	41 (14; 67)
*Female*				
65–74	3.3	1.1	2.2 (1.3; 3.1)	125 (73; 177)
75–84	5.7	3.3	2.4 (1.4; 3.4)	107 (62; 153)
85+	13.3	7.7	5.6 (3.8; 7.5)	102 (68; 136)

*Note*: As the prevented number of hospitalizations for each age group and sex was separately estimated using different models, the sum of each strata value doesn´t correspond to the total number of prevented hospitalizations.

### Control conditions and sensitivity analysis

No changes in trends of other specific bacterial pneumonia hospitalization rate were found when pre-PCV7 and pre-PCV13 periods were compared to PCV7 and PCV13 periods (S1–S4 Tables, S1–S4 Figs).

Decreasing trends in hospitalization rates were verified when alternative PP outcomes definitions (PP in first seven diagnoses or PP combined with sepsis and PP) were used (S5–S8 Tables, S5–S8 Figs).

## Discussion

During the 18 years comprised in this study we observed a steadily reduction of PP hospitalization rate in elderly following PCV13 use and significant reduction in closely years after PCV7 commercialization in some population subgroups. To our knowledge this is one of the first European studies that reports an indirect effect of PCV13 on elderly PP hospitalizations, after several years of low-medium PCV7 coverage. Thus, supporting the hypothesis of the indirect effect of PCV on pneumococcal disease in elderly [6]. The lack of similar effect on other bacterial pneumonia also supports this hypothesis as if PP hospitalization decreasing occurred due to changes in other common risk factors of pneumonia, a similar reduction should be observed in other bacterial pneumonia hospitalization rate. Low vaccine coverage achieved in elderly during the study period make also implausible that such reduction could be mainly attributed to the direct effect of recommended pneumococcal vaccines for this age group.

After PCV13 use, PP hospitalization rate declined by 11% per year, meaning that in 2015/16 approximately, 600 PP hospitalizations were prevented among those aged 65 years and more by PCV13 childhood vaccination, even after several years of PCV7 use. These results strongly suggest that PCV13 use might have quickly reduced the high burden of PP caused by the 6 additional serotypes included in PCV13 [29]. Differences in PCV7 and PCV13 coverage are unlikely to explain our results, as vaccine shift was not accompanied by a great vaccine coverage increase.

Similar results were reported by Simonsen et al [10] in USA, but our absolute rate difference over raised their results, probably because Portuguese PP hospitalizations in pre-PCV13 period almost doubled USA figures. The opposite findings reported in Scotland and England were attributed to potential undercoding of PP hospitalization [17] and non-vaccine serotype replacement in elderly [18].

Whilst major annual relative reduction was estimated between 65 and 74 years, for both male and female, PP hospitalization absolute reduction attributed to PCV13 increased with age.

We were unable to include chronic co-morbidities information within our study group given the impossibility to gather accurate data. However, differences in co-morbidities prevalence expected by age group [30] might have played a key role on the observed differences. Mainly because patients with chronic conditions are more prone to have more severe infections due to serotypes with low invasive potential, that are not included in vaccines. Therefore pneumococci carriage prevalence and circulating serotypes may differ according to the chronic conditions prevalence and a smaller indirect effect of PCV13 is expected in population with high prevalence of chronic conditions [9]. Nevertheless, since age is a well-known risk factor of PP [31], it is not surprisingly that older age groups are those with higher burden of PP hospitalization and consequently those who have higher potential for prevention [9], even considering that the relative reduction after PCV13 vaccine was greater in young seniors than in the older ones. Similar results were previously found in Finland regarding to all-cause pneumonia and PP as well [16].

Between 2002 and 2005, we found statistically significant reduction in PP hospitalization rate in overall translating in less 809 PP hospitalizations than expected in this population in 2004/05. Time delays in the indirect effect of the vaccines have been previously reported [9], meaning PCV7 impact could be greater after a few years of PCV7 use [6,32,33]. Unfortunately we couldn’t estimate a more delayed effect of the PCV7 effect due the low number of years in pre-PCV7.The importance of achieving high coverage rates to obtain herd protection for pneumonia and IPD is highlighted in literature [6,9], which may explained the absence of effect observed in some age groups.

The reduced span of available data in pre-PCV7 period limited the length of the studied PCV7 period, restricting the possibility to quantify the expected PCV7 delayed impact. However, between July 2004 and December 2009, PP hospitalization rate declined by 4% per year, indicating a more delayed impact of PCV7, which is in accordance with serotype circulation figures described by Horácio et al between 1999 and 2011 [13].

Owing to the fact that in our time-series PP hospitalizations correspond only to 5.1% of all-cause pneumonia hospitalizations, a much lower value than the pneumonia etiological fraction usually reported to be associated with *Streptococcus pneunoniae*, we believe that our results underestimated the impact of PCV and represents only the “tip of iceberg”. Some authors [16] overcame this limitation using all-cause pneumonia as outcome. However, all-cause pneumonia may be greatly impacted by other respiratory agents outbreaks [34] and epidemics [8] that cannot be prevented by PCV, which will strongly dilute the PCV effect. All-cause pneumonia it is particularly affected by influenza epidemics, which vary season by season [27]. Therefore, it doesn´t seem plausible the reduction observed in all-cause pneumonia might be attributable PCV, and we consider that using a more specific outcome, PCV indirect effect estimates were less prone to be biased by factors impossible to control.

Influenza-like illness activity was introduced in the Poisson regression model to control for potential seasonal influenza confounding, similar adjustment was performed in few other studies [8,10] and may be considered a strength of this study as seasonality terms typically used in similar analytic models may not control for the influenza severity variability across seasons that differently impacts PP hospitalizations.

Ours study results should be interpreted considering the potential limitations related to the use of a hospital discharge registry mainly used for reimbursement proposes. Changes in admission guidelines, diagnosis and coding practices might be probable sources of bias. Smith et al [35] hypothesized that the reduction of pneumonia case severity after vaccination may have resulted in less frequent use of specific codes, since etiological diagnosis might be associated to more severe cases. However, considering the increasing availability of the rapid urine antigen test for *Streptococcus pneumoniae* within Portuguese hospitals for non-bacteremic PP diagnosis, it is unlikely that diagnosis changes may explain the observed reduction of PP hospitalizations. Furthermore, similar changes in other bacterial pneumonia hospitalizations were not observed.

Changes in antimicrobial resistance and in outpatients’ treatment frequency might altered PP hospitalization rate. However, we considered that those changes are unlike to explain the observed reduction, as the increasing frequency of antimicrobial resistance and the growing number of elderly people living alone (which is a criterion for pneumonia hospitalization) would contributed to the opposite trend.

Another source of bias came from private hospitals, which were not included in hospital discharge registry, but had been progressively integrated in the healthcare system, especially in urban areas during the last decade [36,37]. We cannot exclude that such fact could contributed to the observed decreasing trend in PP hospitalizations but it is not plausible that such effect occurred preferentially in PP rather than in all-cause hospitalizations and in other bacterial pneumonia hospitalizations.

### Conclusion

These results showing a decrease in PP hospitalization rate in elderly after PCV13 use support the indirect effect hypothesis of PCV13 child vaccination even after several years of PCV7 use. The ageing of the population highlights the importance of this indirect effect on PP prevention.

This effect may be a few years delayed regarding vaccine use but it may also be affected by non-vaccine serotype replacement, being critical to monitor pneumococcal disease burden in next years. A full assessment of benefits of PCV13 achieved after its introduction on NIP required further studies with clinical and microbiological data integration.

## Supporting information

S1 Table Other specific bacterial pneumonias hospitalization rate per 10,000 inhabitants stratified by sex and age group between 1998/99 and 2015/16, Portugal mainland. (DOCX)

S2 Table Annual trends of other specific bacterial pneumonias hospitalization rate by sex and age group before and after PCV7 and PCV13 use, Portugal mainland. (DOCX)

S3 Table All-cause hospitalization rate per 10,000 inhabitants stratified by sex and age group, between 1998/99 and 2015/16, Portugal mainland. (DOCX)

S4 Table Annual trends of all-cause hospitalization rate by sex and age group, before and after PCV7 and PCV13 use, Portugal mainland. (DOCX)

S5 Table Hospitalization rate per 10,000 inhabitants of PP in primary diagnosis combined with sepsis/bacteremia in first diagnosis and PP in secondary diagnosis between 1998/99 and 2015/16, Portugal mainland. (DOCX)

S6 Table Annual trends of PP combined with sepsis and PP hospitalization rate by sex and age group, before and after PCV7 and PCV13 introduction, Portugal mainland. (DOCX)

S7 Table Hospitalization rate per 10,000 inhabitants of PP in first seven diagnoses between 1998/99 and 2015/16, Portugal mainland. (DOCX)

S8 Table Annual trends of PP in first seven diagnoses hospitalization rate by sex and age group, before and after PCV7 and PCV13 introduction, Portugal mainland. (DOCX)

S1 Fig Trends in bacterial pneumonia hospitalizations by sex and age group before and after PCV7 introduction. (TIF)

S2 Fig Trends in bacterial pneumonia hospitalizations by sex and age group before and after PCV13 introduction. (TIF)

S3 Fig Trends of all-cause hospitalizations by sex and age group, before and after PCV7 introduction. (TIF)

S4 Fig Trends of all-cause hospitalizations by sex and age group, before and after PCV13 introduction. (TIF)

S5 Fig Trends in PP combined with sepsis and PP hospitalizations by sex and age group, before and after PCV7 introduction. (TIF)

S6 Fig Trends in PP combined with sepsis and PP hospitalizations by sex and age group before and afterPCV13 introduction. (TIF)

S7 Fig Trends in PP (in first seven diagnoses) hospitalizations by sex and age group, before and after PCV7 introduction. (TIF)

S8 Fig Trends in PP (in first seven diagnoses) hospitalizations by sex and age group, before and after PCV13 introduction. (TIF)
