# Supplementary material for: Indirect effect of 7-valent and 13-valent pneumococcal conjugated vaccines on pneumococcal pneumonia hospitalizations in elderly
Source: PLoS One. 2019 Jan 16;14(1):e0209428. doi: 10.1371/journal.pone.0209428 (PMC6334925; doi:10.1371/journal.pone.0209428)
Supplement: S1 Table — (DOCX) [file pone.0209428.s001.docx]

**S1 Table.** Other specific bacterial pneumonias hospitalization rate per 10,000 inhabitants stratified by sex and age group between 1998/99 and 2015/16, Portugal mainland.

| **Year** | **Total** | **Male** | | | **Female** | | |
| --- | --- | --- | --- | --- | --- | --- | --- |
|  |  | **65-74** | **75-84** | **85+** | **65-74** | **75-84** | **85+** |
| **1998/99** | 2.2 | 2.2 | 4.8 | 8.1 | 0.9 | 1.6 | 2.5 |
| **1999/00** | 2.5 | 2.5 | 5.1 | 11.0 | 1.0 | 2.2 | 2.5 |
| **2000/01** | 3.0 | 3.1 | 6.0 | 11.2 | 0.9 | 2.5 | 4.6 |
| **2001/02** | 3.0 | 3.3 | 5.5 | 10.6 | 1.1 | 2.3 | 5.5 |
| **2002/03** | 3.0 | 2.8 | 6.8 | 13.7 | 0.7 | 2.3 | 5.0 |
| **2003/04** | 4.1 | 3.6 | 9.2 | 16.2 | 1.2 | 3.6 | 6.2 |
| **2004/05** | 3.6 | 3.2 | 7.5 | 18.7 | 0.7 | 2.9 | 6.5 |
| **2005/06** | 3.2 | 2.9 | 7.2 | 15.7 | 0.8 | 2.1 | 5.8 |
| **2006/07** | 3.9 | 2.4 | 8.8 | 18.1 | 1.1 | 3.5 | 6.3 |
| **2007/08** | 3.5 | 2.3 | 7.6 | 15.8 | 1.0 | 2.8 | 6.3 |
| **2008/09** | 4.0 | 2.6 | 9.3 | 18.8 | 0.9 | 2.8 | 8.0 |
| **2009/10** | 4.0 | 2.1 | 9.2 | 19.0 | 0.9 | 3.2 | 7.2 |
| **2010/11** | 4.3 | 3.2 | 8.2 | 20.5 | 1.0 | 3.3 | 8.2 |
| **2011/12** | 4.2 | 3.0 | 7.7 | 21.5 | 0.8 | 3.2 | 8.1 |
| **2012/13** | 4.1 | 2.5 | 8.5 | 20.2 | 0.8 | 2.7 | 7.8 |
| **2013/14** | 4.3 | 2.9 | 7.8 | 21.3 | 1.0 | 2.9 | 8.6 |
| **2014/15** | 4.0 | 3.0 | 7.3 | 18.8 | 0.8 | 2.8 | 7.1 |
| **2015/16** | 4.7 | 3.0 | 9.2 | 23.1 | 1.0 | 2.9 | 8.9 |
