# Supplementary material for: Indirect effect of 7-valent and 13-valent pneumococcal conjugated vaccines on pneumococcal pneumonia hospitalizations in elderly
Source: PLoS One. 2019 Jan 16;14(1):e0209428. doi: 10.1371/journal.pone.0209428 (PMC6334925; doi:10.1371/journal.pone.0209428)
Supplement: S2 Table — (DOCX) [file pone.0209428.s002.docx]

**S2 Table.** Annual trends of other specific bacterial pneumonias hospitalization rate by sex and age group before and after PCV7 and PCV13 use, Portugal mainland.

|  | **Pre-PCV study period** | | **PCV study period** | | **Test for change in trend** |
| --- | --- | --- | --- | --- | --- |
|  | **RR** | **95% CI** | **RR** | **95% CI** |  |
| **PCV7** |  | | | |  |
| **Total** | 1.11 | (1.04; 1.17) | 1.05 | (0.92; 1.19) | 0.456 |
| ***Male*** |  | | | |  |
| **65-74** | 1.13 | (1.02; 1.27) | 0.85 | (0.68; 1.07) | 0.178 |
| **75-84** | 1.13 | (1.02; 1.26) | 1.05 | (0.84; 1.29) | 0.662 |
| **85+** | 1.11 | (0.93; 1.33) | 1.20 | (0.84; 1.72) | 0.341 |
| ***Female*** |  | | | |  |
| **65-74** | 0.98 | (0.84; 1.16) | 1.09 | (0.77; 1.56) | 0.609 |
| **75-84** | 1.06 | (0.90; 1.24) | 1.21 | (0.88; 1.68) | 0.211 |
| **85+** | 1.24 | (1.00; 1.54) | 0.95 | (0.62; 1.46) | 0.861 |
| **PCV13** |  | | | |  |
| **Total** | 1.01 | (1.00; 1.03) | 1.00 | (0.96; 1.03) | 0.797 |
| ***Male*** |  | | | |  |
| **65-74** | 1.00 | (0.95; 1.04) | 1.02 | (0.94; 1.12) | 0.593 |
| **75-84** | 1.00 | (0.97; 1.04) | 1.00 | (0.93; 1.08) | 0.950 |
| **85+** | 1.02 | (0.97; 1.07) | 1.02 | (0.93; 1.11) | 0.698 |
| ***Female*** |  | | | |  |
| **65-74** | 1.01 | (0.94; 1.08) | 1.00 | (0.86; 1.16) | 0.999 |
| **75-84** | 1.05 | (1.00; 1.09) | 0.92 | (0.84; 1.00) | 0.058 |
| **85+** | 1.03 | (0.98; 1.08) | 1.00 | (0.91; 1.10) | 0.989 |
