# Supplementary material for: Indirect effect of 7-valent and 13-valent pneumococcal conjugated vaccines on pneumococcal pneumonia hospitalizations in elderly
Source: PLoS One. 2019 Jan 16;14(1):e0209428. doi: 10.1371/journal.pone.0209428 (PMC6334925; doi:10.1371/journal.pone.0209428)
Supplement: S3 Table — (DOCX) [file pone.0209428.s003.docx]

**S3 Table.** All-cause hospitalization rate per 10,000 inhabitants stratified by sex and age group, between 1998/99 and 2015/16, Portugal mainland.

| **Year** | **Total** | **Male** | | | **Female** | | |
| --- | --- | --- | --- | --- | --- | --- | --- |
|  |  | **65-74** | **75-84** | **85+** | **65-74** | **75-84** | **85+** |
| **1998/99** | 1629.9 | 1656.3 | 2236.4 | 2909.4 | 1154.7 | 1648.7 | 2238.9 |
| **1999/00** | 1596.1 | 1624.4 | 2196.1 | 2655.9 | 1131.1 | 1630.8 | 2120.7 |
| **2000/01** | 1627.5 | 1640.5 | 2258.6 | 2597.5 | 1140.1 | 1714.1 | 2052.6 |
| **2001/02** | 1671.6 | 1663.5 | 2315.0 | 2722.1 | 1149.5 | 1773.0 | 2200.4 |
| **2002/03** | 1689.4 | 1654.9 | 2350.8 | 2744.3 | 1174.9 | 1781.0 | 2252.0 |
| **2003/04** | 1729.7 | 1665.3 | 2422.7 | 2875.0 | 1188.1 | 1830.5 | 2381.6 |
| **2004/05** | 1683.2 | 1603.9 | 2361.7 | 2850.2 | 1141.7 | 1782.4 | 2371.4 |
| **2005/06** | 1662.0 | 1581.7 | 2334.2 | 2858.1 | 1132.1 | 1730.4 | 2296.8 |
| **2006/07** | 1662.5 | 1558.0 | 2319.5 | 2880.5 | 1117.7 | 1728.6 | 2354.7 |
| **2007/08** | 1666.2 | 1557.0 | 2291.6 | 2892.2 | 1112.8 | 1703.4 | 2453.9 |
| **2008/09** | 1702.6 | 1580.9 | 2319.6 | 3057.8 | 1114.8 | 1729.1 | 2576.5 |
| **2009/10** | 1652.6 | 1519.8 | 2250.3 | 3032.1 | 1082.2 | 1656.2 | 2507.1 |
| **2010/11** | 1640.1 | 1504.9 | 2226.0 | 2945.6 | 1057.5 | 1659.0 | 2428.7 |
| **2011/12** | 1656.0 | 1507.8 | 2214.4 | 3061.7 | 1058.6 | 1653.2 | 2500.5 |
| **2012/13** | 1671.2 | 1501.4 | 2234.3 | 3111.5 | 1062.7 | 1659.0 | 2568.2 |
| **2013/14** | 1672.0 | 1486.8 | 2240.1 | 3241.0 | 1031.5 | 1649.2 | 2642.7 |
| **2014/15** | 1639.2 | 1452.8 | 2162.5 | 3236.5 | 988.4 | 1618.4 | 2666.8 |
| **2015/16** | 1643.0 | 1451.3 | 2192.1 | 3261.8 | 983.6 | 1615.5 | 2636.8 |

***Note:*** All-cause pneumonia hospitalizations not included.
