# Supplementary material for: Indirect effect of 7-valent and 13-valent pneumococcal conjugated vaccines on pneumococcal pneumonia hospitalizations in elderly
Source: PLoS One. 2019 Jan 16;14(1):e0209428. doi: 10.1371/journal.pone.0209428 (PMC6334925; doi:10.1371/journal.pone.0209428)
Supplement: S4 Table — (DOCX) [file pone.0209428.s004.docx]

**S4 Table.** Annual trends of all-cause hospitalization rate by sex and age group, before and after PCV7 and PCV13 use, Portugal mainland.

|  | **Pre-PCV study period** | | **PCV study period** | | **Test for change in trend** |
| --- | --- | --- | --- | --- | --- |
|  | **RR** | **95% CI** | **RR** | **95% CI** |  |
| **PCV7** |  | | | |  |
| **Total** | 1.01 | (1.00; 1.02) | 1.01 | (0.98; 1.03) | 0.562 |
| ***Male*** |  | | | |  |
| **65-74** | 1.00 | (0.98; 1.03) | 1.00 | (0.95; 1.06) | 0.922 |
| **75-84** | 1.02 | (1.01; 1.04) | 0.99 | (0.95; 1.04) | 0.893 |
| **85+** | 0.99 | (0.97; 1.01) | 1.04 | (0.98; 1.09) | 0.129 |
| ***Female*** |  | | | |  |
| **65-74** | 1.00 | (0.98; 1.03) | 1.01 | (0.97; 1.06) | 0.700 |
| **75-84** | 1.03 | (1.02; 1.05) | 0.99 | (0.95; 1.03) | 0.512 |
| **85+** | 1.01 | (0.99; 1.03) | 1.04 | (1.00; 1.09) | 0.074 |
| **PCV13** |  | | | |  |
| **Total** | 0.99 | (0.98; 1.00) | 1.00 | (0.99; 1.01) | 0.663 |
| ***Male*** |  | | | |  |
| **65-74** | 0.99 | (0.98; 1.00) | 1.00 | (0.99; 1.01) | 0.947 |
| **75-84** | 0.99 | (0.98; 1.00) | 1.00 | (0.99; 1.01) | 0.492 |
| **85+** | 1.01 | (1.00; 1.02) | 1.01 | (0.99; 1.02) | 0.114 |
| ***Female*** |  | | | |  |
| **65-74** | 0.99 | (0.98; 1.00) | 0.98 | (0.97; 1.00) | 0.214 |
| **75-84** | 0.99 | (0.98; 1.00) | 1.00 | (0.99; 1.01) | 0.831 |
| **85+** | 1.01 | (1.00; 1.02) | 1.00 | (0.99; 1.01) | 0.949 |
