# Supplementary material for: Indirect effect of 7-valent and 13-valent pneumococcal conjugated vaccines on pneumococcal pneumonia hospitalizations in elderly
Source: PLoS One. 2019 Jan 16;14(1):e0209428. doi: 10.1371/journal.pone.0209428 (PMC6334925; doi:10.1371/journal.pone.0209428)
Supplement: S5 Table — (DOCX) [file pone.0209428.s005.docx]

**S5 Table.** Hospitalization rate per 10,000 inhabitants of PP in primary diagnosis combined with sepsis/bacteremia in first diagnosis and PP in secondary diagnosis between 1998/99 and 2015/16, Portugal mainland.

| **Year** | **Total** | **Male** | | | **Female** | | |
| --- | --- | --- | --- | --- | --- | --- | --- |
|  |  | **65-74** | **75-84** | **≥ 85** | **65-74** | **75-84** | **≥ 85** |
| 1998/99 | 7.0 | 6.3 | 12.6 | 20.7 | 3.2 | 6.5 | 14.5 |
| 1999/00 | 7.3 | 5.1 | 13.5 | 28.5 | 3.4 | 7.8 | 13.4 |
| 2000/01 | 8.0 | 6.9 | 13.1 | 30.3 | 3.3 | 8.4 | 14.8 |
| 2001/02 | 9.5 | 6.9 | 17.5 | 34.0 | 4.3 | 8.7 | 20.9 |
| 2002/03 | 8.7 | 6.7 | 15.1 | 34.6 | 3.8 | 8.3 | 19.0 |
| 2003/04 | 10.3 | 7.2 | 18.8 | 38.6 | 4.0 | 9.8 | 23.7 |
| 2004/05 | 9.0 | 6.5 | 16.3 | 39.9 | 3.1 | 8.8 | 18.8 |
| 2005/06 | 7.5 | 5.5 | 14.8 | 29.1 | 2.5 | 6.9 | 15.7 |
| 2006/07 | 8.2 | 6.0 | 14.7 | 29.9 | 3.1 | 7.2 | 18.6 |
| 2007/08 | 7.8 | 5.6 | 13.2 | 30.1 | 2.2 | 6.8 | 21.2 |
| 2008/09 | 9.2 | 6.6 | 15.8 | 31.2 | 3.3 | 8.3 | 21.3 |
| 2009/10 | 7.2 | 5.4 | 11.7 | 28.3 | 2.3 | 6.5 | 16.1 |
| 2010/11 | 7.1 | 5.4 | 11.2 | 25.8 | 2.6 | 5.9 | 15.2 |
| 2011/12 | 7.2 | 4.3 | 11.2 | 28.9 | 2.4 | 6.2 | 17.7 |
| 2012/13 | 4.4 | 3.7 | 6.9 | 16.8 | 1.2 | 3.7 | 9.5 |
| 2013/14 | 4.7 | 3.4 | 6.6 | 16.9 | 1.3 | 4.3 | 11.8 |
| 2014/15 | 4.8 | 3.2 | 7.1 | 18.2 | 1.6 | 4.1 | 9.9 |
| 2015/16 | 4.5 | 3.0 | 6.6 | 17.4 | 1.4 | 4.2 | 9.3 |

***Note:*** *Sepsis/bacteremia in primary diagnosis (CID9-CM: 038, 995.91, 995.92, 785.92, 790.7; CID10-CM: A40, A41, R65.20, R65.21, R78.81.) and ACP as secondary diagnosis (up to six diagnoses); ********Sepsis/bacteremia in primary diagnosis (CID9-CM: 038, 995.91, 995.92, 785.92, 790.7; CID10-CM: A40, A41, R65.20, R65.21, R78.81) and PP as secondary diagnosis (up to six diagnoses).
