# Supplementary material for: Indirect effect of 7-valent and 13-valent pneumococcal conjugated vaccines on pneumococcal pneumonia hospitalizations in elderly
Source: PLoS One. 2019 Jan 16;14(1):e0209428. doi: 10.1371/journal.pone.0209428 (PMC6334925; doi:10.1371/journal.pone.0209428)
Supplement: S6 Table — (DOCX) [file pone.0209428.s006.docx]

**S6 Table.** Annual trends of PP combined with sepsis and PP hospitalization rate by sex and age group, before and after PCV7 and PCV13 introduction, Portugal mainland.

|  | **Pre-PCV study period** | | **PCV study period** | | | **Test for change in trend** | |
| --- | --- | --- | --- | --- | --- | --- | --- |
|  | **RR** | **95% CI** | **RR** | **95% CI** | |  |  |
| **PCV7** |  | | | |  | |  |
| **Total** | **1.14** | (1.10; 1.20) | 0.92 | (0.84; 1.01) | | 0.060 | |
| ***Male*** |  | | | |  | |  |
| **65-74** | **1.13** | (1.02; 1.25) | 0.87 | (0.69, 1.08) | | 0.207 | |
| **75-84** | **1.15** | (1.04; 1.27) | 0.98 | (0.79; 1.21) | | 0.838 | |
| **85+** | **1.17** | (1.02; 1.31) | 0.93 | (0.71; 1.21) | | 0.584 | |
| ***Female*** |  | | | |  | |  |
| **65-74** | **1.16** | (1.06; 1.27) | 0.84 | (0.69, 1.02) | | 0.076 | |
| **75-84** | **1.15** | (1.05; 1.25) | 0.88 | (0.73, 1.05) | | 0.164 | |
| **85+** | **1.16** | (1.01; 1.33) | 0.98 | (0.74; 1.29) | | 0.858 | |
| **PCV13** |  | | | |  | |  |
| **Total** | **0.96** | (0.95; 0.98) | **0.90** | (0.87, 0.93) | | <0.001 | |
| ***Male*** |  | | | |  | |  |
| **65-74** | 0.97 | (0.94; 1.01) | **0.87** | (0.81, 0.95) | | 0.001 | |
| **75-84** | **0.95** | (0.92; 0.98) | **0.91** | (0.84; 0.98) | | 0.014 | |
| **85+** | **0.95** | (0.91; 0.99) | 0.95 | (0.88; 1.04) | | 0.267 | |
| ***Female*** |  | | | |  | |  |
| **65-74** | 0.99 | (0.94; 1.04) | **0.82** | (0.73, 0.93) | | 0.001 | |
| **75-84** | **0.95** | (0.92; 0.99) | **0.92** | (0.84; 0.99) | | 0.037 | |
| **85+** | 0.97 | (0.94; 1.01) | **0.90** | (0.84, 0.97) | | 0.008 | |

***Note:*** Statistically significant values are represented in bold.
