# Supplementary material for: Indirect effect of 7-valent and 13-valent pneumococcal conjugated vaccines on pneumococcal pneumonia hospitalizations in elderly
Source: PLoS One. 2019 Jan 16;14(1):e0209428. doi: 10.1371/journal.pone.0209428 (PMC6334925; doi:10.1371/journal.pone.0209428)
Supplement: S7 Table — (DOCX) [file pone.0209428.s007.docx]

**S7 Table.** Hospitalization rate per 10,000 inhabitants of PP in first seven diagnoses between 1998/99 and 2015/16, Portugal mainland.

| **Year** | **Total** | **Male** | | | **Female** | | |
| --- | --- | --- | --- | --- | --- | --- | --- |
|  |  | **65-74** | **75-84** | **≥ 85** | **65-74** | **75-84** | **≥ 85** |
| **1998/99** | 9.4 | 8.5 | 17.6 | 27.3 | 4.3 | 8.5 | 18.8 |
| **1999/00** | 9.8 | 7.2 | 19.2 | 36.2 | 4.4 | 10.1 | 17.0 |
| **2000/01** | 10.7 | 9.5 | 18.4 | 38.9 | 4.2 | 10.8 | 19.1 |
| **2001/02** | 12.7 | 9.7 | 22.9 | 43.1 | 5.7 | 12.0 | 26.8 |
| **2002/03** | 11.5 | 9.0 | 19.9 | 43.5 | 4.9 | 11.1 | 24.4 |
| **2003/04** | 12.8 | 9.7 | 23.8 | 47.8 | 5.0 | 11.8 | 28.1 |
| **2004/05** | 11.6 | 8.6 | 21.6 | 51.1 | 4.0 | 11.1 | 23.5 |
| **2005/06** | 9.8 | 7.3 | 18.8 | 37.8 | 3.5 | 8.8 | 20.1 |
| **2006/07** | 10.5 | 7.8 | 18.2 | 39.8 | 3.9 | 9.3 | 25.0 |
| **2007/08** | 10.3 | 7.3 | 17.4 | 40.7 | 3.1 | 8.9 | 27.6 |
| **2008/09** | 11.2 | 8.1 | 19.7 | 38.7 | 3.8 | 10.1 | 25.2 |
| **2009/10** | 8.9 | 6.9 | 14.9 | 33.7 | 2.7 | 7.8 | 19.5 |
| **2010/11** | 8.5 | 6.7 | 13.2 | 30.2 | 3.2 | 7.2 | 18.1 |
| **2011/12** | 8.7 | 5.5 | 13.4 | 34.5 | 2.9 | 7.4 | 21.0 |
| **2012/13** | 5.2 | 4.3 | 8.2 | 20.0 | 1.7 | 4.1 | 10.5 |
| **2013/14** | 5.3 | 3.9 | 7.7 | 18.1 | 1.5 | 4.8 | 12.5 |
| **2014/15** | 5.4 | 4.0 | 8.2 | 20.7 | 1.7 | 4.7 | 10.9 |
| **2015/16** | 5.1 | 3.7 | 7.4 | 18.8 | 1.6 | 4.8 | 10.3 |
