# Supplementary material for: Indirect effect of 7-valent and 13-valent pneumococcal conjugated vaccines on pneumococcal pneumonia hospitalizations in elderly
Source: PLoS One. 2019 Jan 16;14(1):e0209428. doi: 10.1371/journal.pone.0209428 (PMC6334925; doi:10.1371/journal.pone.0209428)
Supplement: S8 Table — (DOCX) [file pone.0209428.s008.docx]

**S8 Table.** Annual trends of PP in first seven diagnoses hospitalization rate by sex and age group, before and after PCV7 and PCV13 introduction, Portugal mainland.

|  | **Pre-PCV study period** | | **PCV study period** | | **Test for change in trend** |
| --- | --- | --- | --- | --- | --- |
|  | **RR** | **95% CI** | **RR** | **95% CI** |  |
| **PCV7** |  | | | |  |
| **Total** | 1.14 | (1.10; 1.18) | **0.90** | (0.83; 0.97) | 0.007 |
| ***Male*** |  | | | |  |
| **65-74** | 1.13 | (1.04; 1.24) | 0.87 | (0.72; 1.05) | 0.143 |
| **75-84** | 1.10 | (1.02; 1.18) | 0.99 | (0.86; 1.15) | 0.933 |
| **85+** | 1.16 | (1.04; 1.30) | 0.94 | (0.74; 1.18) | 0.570 |
| ***Female*** |  | | | |  |
| **65-74** | 1.16 | (1.07; 1.25) | **0.82** | (0.69; 0.97) | 0.018 |
| **75-84** | 1.17 | (1.08; 1.25) | **0.81** | (0.70; 0.95) | 0.008 |
| **85+** | 1.15 | (1.02; 1.31) | 0.95 | (0.74; 1.23) | 0.711 |
| **PCV13** |  | | | |  |
| **Total** | **0.95** | (0.94; 0.97) | **0.89** | (0.86; 0.92) | 0.95 |
| ***Male*** |  | | | |  |
| **65-74** | **0.95** | (0.93; 0.98) | **0.90** | (0.84; 0.96) | 0.95 |
| **75-84** | **0.94** | (0.91; 0.97) | **0.89** | (0.83; 0.96) | 0.94 |
| **85+** | **0.94** | (0.91; 0.98) | 0.93 | (0.86; 1.01) | 0.94 |
| ***Female*** |  | | | |  |
| **65-74** | 0.98 | (0.93; 1.02) | **0.82** | (0.74; 0.92) | 0.98 |
| **75-84** | **0.95** | (0.91; 0.98) | **0.90** | (0.84; 0.98) | 0.012 |
| **85+** | **0.97** | (0.94; 1.00) | **0.88** | (0.82; 0.94) | 0.000 |

***Note:*** Statistically significant values are represented in bold.
