# Supplementary figures and images for: Indirect effect of 7-valent and 13-valent pneumococcal conjugated vaccines on pneumococcal pneumonia hospitalizations in elderly
Source: PLoS One. 2019 Jan 16;14(1):e0209428. doi: 10.1371/journal.pone.0209428 (PMC6334925; doi:10.1371/journal.pone.0209428)

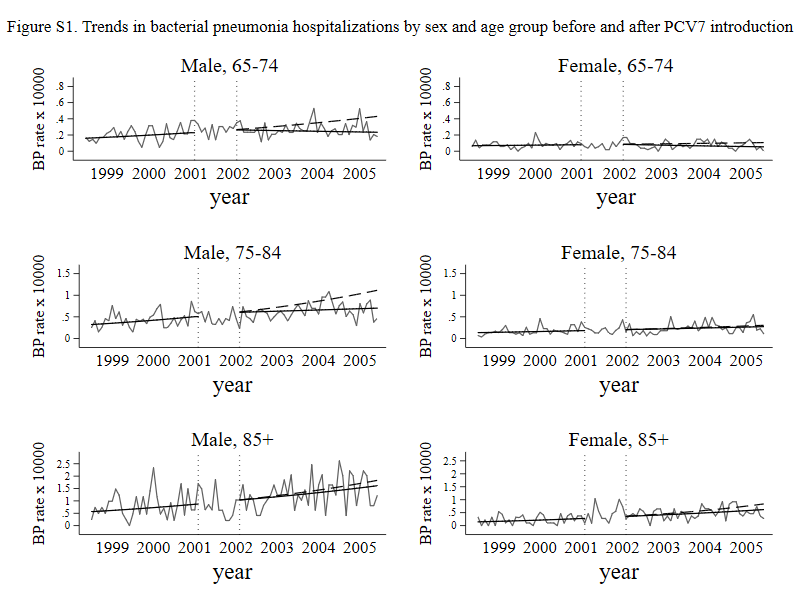

Supplement: S1 Fig — (TIF) [file pone.0209428.s009.tif]

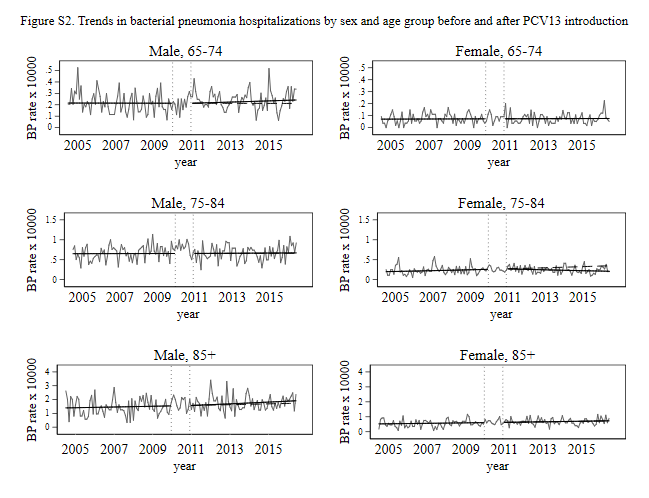

Supplement: S2 Fig — (TIF) [file pone.0209428.s010.tif]

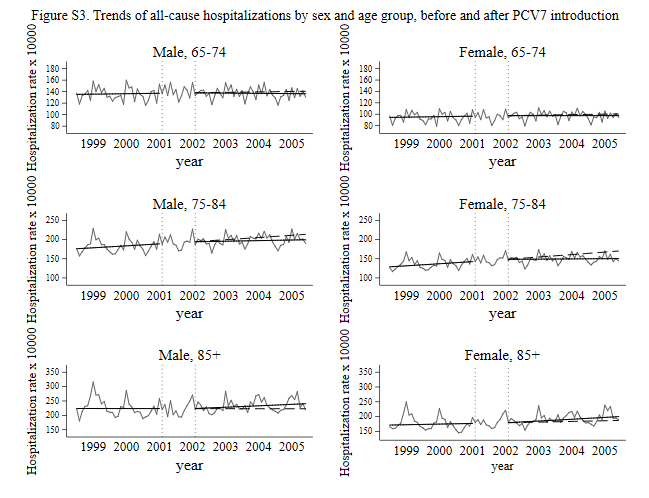

Supplement: S3 Fig — (TIF) [file pone.0209428.s011.tif]

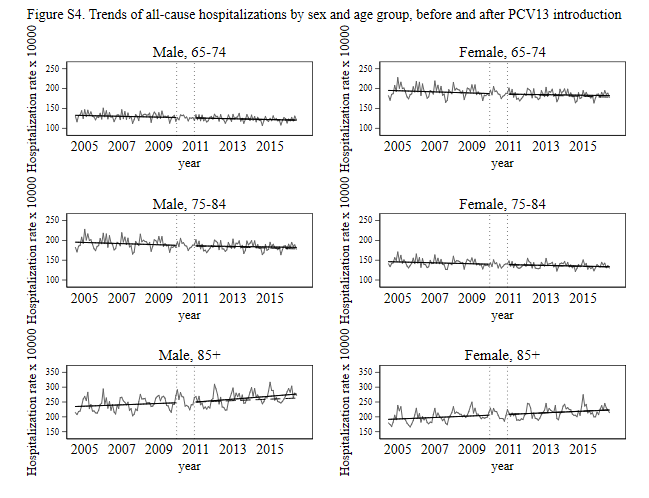

Supplement: S4 Fig — (TIF) [file pone.0209428.s012.tif]

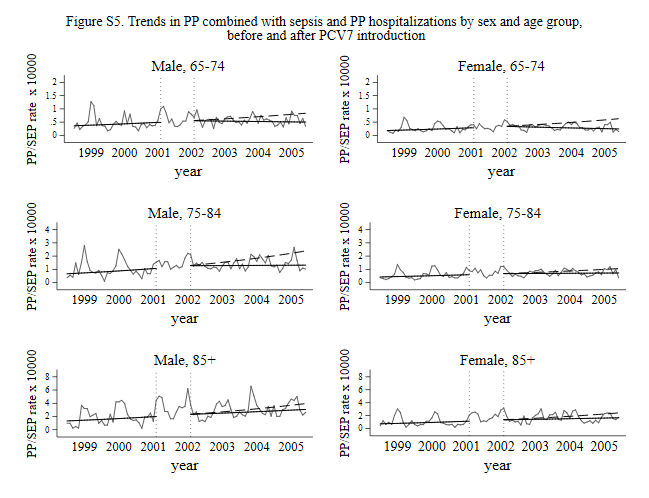

Supplement: S5 Fig — (TIF) [file pone.0209428.s013.tif]

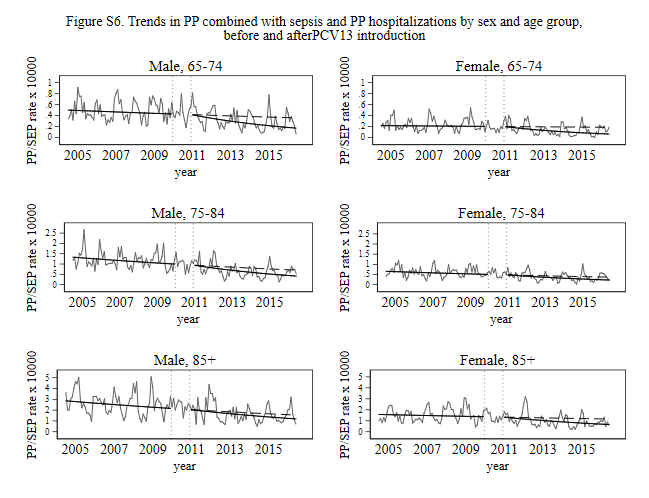

Supplement: S6 Fig — (TIF) [file pone.0209428.s014.tif]

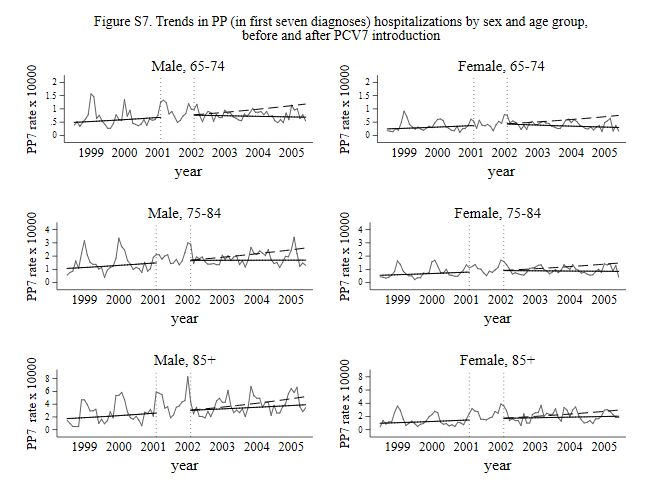

Supplement: S7 Fig — (TIF) [file pone.0209428.s015.tif]

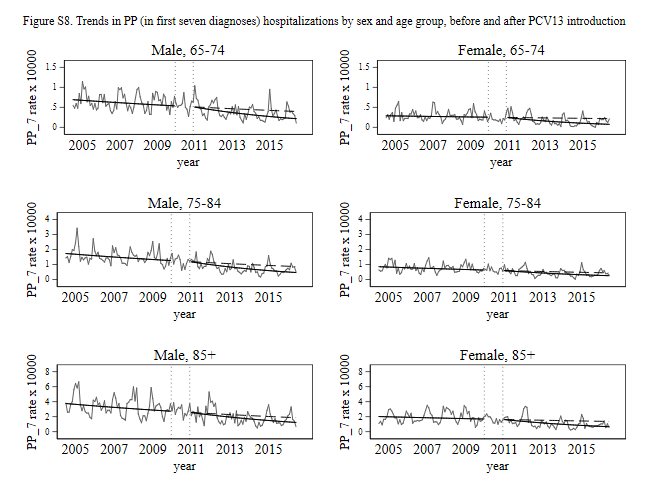

Supplement: S8 Fig — (TIF) [file pone.0209428.s016.tif]
